# Correlation of geopolitics, education, democracy with COVID-19 vaccination rate

**DOI:** 10.1186/s12889-024-18215-4

**Published:** 2024-03-04

**Authors:** Konstantine Chakhunashvili, Davit G. Chakhunashvili, Eka Kvirkvelia, Tornike Toria, Liza Basilaia, Tsira Gorjomeladze

**Affiliations:** 1 The University of Georgia, Tbilisi, Georgia; 2 Reproductive Education Hub, Tbilisi, Georgia; 3 Department of Pediatrics, Alte University, Tbilisi, Georgia; 4 Department of Gynecology, Caucasus University, Tbilisi, Georgia; 5 Business and Technology University, Tbilisi, Georgia; 6 Ilia State University, Tbilisi, Georgia

**Keywords:** COVID-19, Patient education, Vaccination rate, Education index, Human development insight, Democracy index

## Abstract

**Introduction:**

Vaccine hesitancy is an ongoing problem and determining the factors that increase the vaccination rate in various countries of the world might be useful for further implementation of efficient public health policies and negating anti-vaccination campaigns.

**Materials and methods:**

Human Development Index (HDI), Education Index (EI), Democracy Index (DI), COVID-19 vaccination rates, COVID-19 data were collected from public sources such as UNDP - Human Development Reports, UNESCO - Education Index, Economist Intelligence, WHO– COVID-19 Dashboard, Our World In Data, The Financial Times COVID-19 Dashboard. Statistical analysis such as Pearson correlation, and linear regression analyses were done to determine a relation between the above-mentioned indices and COVID-19 vaccination rates (1-dose, 2-dose, booster, and combined).

**Results:**

HDI had the strongest positive correlation with the vaccination rates (1-dose– r (181) = 0.632, *p* < 0.001, 2-dose– r (181) = 0.671, *p* < 0.001, booster– r (181) = 0.718, *p* < 0.001, combined– 0.703, *p* < 0.001). EI (1-dose– r (177) = 0.560, *p* < 0.001, 2-dose– r (177) = 0.599, *p* < 0.001, booster– r (177) = 0.642, *p* < 0.001, combined– 0.626, *p* < 0.001), DI (1-dose– r (163) = 0.445, *p* < 0.001, 2-dose– r (163) = 0.479, *p* < 0.001, booster– r (163) = 0.534, *p* < 0.001, combined– 0.508, *p* < 0.001), as well as Geographic location (1-dose– η (Eta) = 0.610 *p* < 0.001, 2-dose– η (Eta) = 0.633 *p* < 0.001, booster– η (Eta) = 0.657, *p* < 0.001, combined– η (Eta) = 0.645, *p* < 0.001) had positive correlation with vaccination rates.

**Conclusion:**

There is a strong positive correlation of COVID-19 vaccination rates with HDI and EI.

**Supplementary Information:**

The online version contains supplementary material available at 10.1186/s12889-024-18215-4.

## Introduction

Vaccines are effective tools in controlling and preventing harmful or deadly infectious diseases, especially in people who are immunocompromised [1]. According to World Health Organization (WHO), between 2010 and 2015, vaccines prevented about 10 million deaths worldwide [1]. Evidence collected throughout the globe suggests that Coronavirus Disease 2019 (COVID-19) vaccines are effective in the reduction of infection rates and prevention of severe illness or death [2]. For the COVID-19 vaccine to achieve the highest efficacy (achieve herd immunity), it is of vital importance that the public vaccination rate is 70% and above [3].

Factors that contribute to the acceptance of vaccination rate may include availability, affordability, effectiveness, and safety of vaccines; or the necessity of vaccination and perception of the disease risk [4]. COVID-19 vaccine hesitancy has been detected in low, middle, and high-income countries globally [5]. According to different data, vaccine refusal in general, including COVID-19 vaccines, is a common phenomenon worldwide and the main reasons for hesitancy involve– a lack of awareness and knowledge, certain religious beliefs, as well a lack of perception of risks or benefits [4]. Cultural, socio-demographic, cognitive, psychological factors, or even political orientations may also lead to vaccine hesitancy [2, 4].

To analyze the real reasons behind the refusal of vaccine acceptance, the epidemiologic triad of host, agent, and environmental factors may be used [4]. Host factors encompass education, income levels, knowledge, or previous experience; The agent factors depend on the perception of susceptibility to the disease, as well as vaccine effectiveness and safety; Environmental factors involve social factors, information spread via media, and public health policy [4]. Distrust of crisis management policy and dissatisfaction with the government policy can influence vaccine hesitancy [5]. On the other hand, strong trust in governments may be a crucial factor for higher rates of vaccine acceptance [6]. Trust in social media or other informal sources of information (friends, family) might negatively affect the vaccination status [7]. Individuals who believe in conspiracy theories, have a high susceptibility to misinformation and are more hesitant to get vaccinated [2]. The conspiracy theories range from COVID-19 being just another flu-like disease to governments reporting fake number of the infected and the dead [8]. In this article we are going to determine which of the factors (geographic location, education, democracy etc.) correlate with the COVID-19 vaccination rate and what is the strength of correlation. This data might be extremely useful for developing novel approaches in public health management.

## Materials and methods

This research has a cross-sectional study design and we tried to detect whether democracy index, geographic location, and level of education are correlated with the COVID-19 vaccination rate, separately, all together or in a various combination. Data collection was performed between June, 2023 and August, 2023. Primary data sources included the United Nation Development Programme, The World Health Organization; Secondary sources included Our World in Data and Financial Times COVID-19 dashboard. No exclusion criteria were used for countries or territories. Healthcare logistics and infrastructure were not a subject data analysis in this study, although it might be the major contributing factor throughout in certain geographic locations.

### Data collection

We collected the following values: Human Development Index (HDI) [9], Education Index (EI) [10], Democracy Index (DI) [11], COVID-19 vaccination rate (1-dose, 2-dose, booster doses) [12–14], COVID-19 death per 1 million (as of November of 2023) [12, 13] [Table 1]. We did not acquire the data from the primary sources, such as local public health authorities, but from secondary trusted sources that acquire and filter the information. The preferred source was the official WHO COVID-19 dashboard, which offers continuous data verification through the official channels to ensure accuracy, reliability, and up-to-date publication of the information. Although, if the data for certain country or territory (for example: Taiwan) was not available through this portal, we utilized Our World in Data or Financial Times COVID-19 dashboards. It should be noted that countries that had vaccination rates more than 100% of the population regarding first or second dose were included with 100%.

**Table 1:** This data depicts all the data that has been collected and analyzed through this study

Country/ Territory	Human development Index	Education Index	Democracy index	1-dose (%)	2-dose (%)	Booster (%)	Combined (%)	Covid-19 death per 1 million population
Afghanistan	0.478	0.415	0.320	29.10	27.30	0.00	56.40	193.61
Albania	0.796	0.745	6.410	47.30	44.77	13.20	105.27	1267.98
Algeria	0.745	0.664	3.660	34.80	17.90	1.30	54.00	153.24
Andorra	0.858	0.714	8.100	76.20	70.20	56.20	202.60	1991.41
Angola	0.586	0.498	3.960	45.30	24.90	4.20	74.40	54
Antigua and Ba	0.788	0.676		65.65	63.70	10.50	139.85	1556.97
Argentina	0.842	0.816	6.850	91.20	83.50	72.60	247.30	2866.87
Armenia	0.759	0.749	5.830	38.10	33.30	1.40	72.80	3155.58
Australia	0.951	0.929	8.710	86.60	84.30	76.70	247.60	903.18
Austria	0.916	0.852	8.200	77.30	74.90	67.40	219.60	2520.81
Azerbaijan	0.745	0.709	2.870	53.60	48.20	34.50	136.30	1000.82
The Bahamas	0.812	0.726	7.900	44.30	42.10	9.30	95.70	2058.59
Bahrain	0.875	0.759	2.520	72.90	72.10	46.10	191.10	1043.31
Bangladesh	0.661	0.508	5.990	90.60	76.30	36.80	203.70	172.19
Barbados	0.790	0.777	0.830	57.00	55.50	30.90	143.40	2105.48
Belarus	0.808	0.838	1.990	69.90	66.20	40.00	176.10	746.52
Belgium	0.937	0.893	7.640	80.20	79.30	62.80	222.30	2946.06
Belize	0.683	0.705	-	63.30	55.60	12.80	131.70	1697.57
Bermuda	-	-	-	-	-	-	-	2569.81
Benin	0.525	0.471	4.280	26.50	22.20	0.00	48.70	12.1
Bhutan	0.666	0.445	5.540	90.60	87.80	82.20	260.60	26.84
Bolivia	0.692	0.687	4.510	63.60	53.40	19.80	136.80	1833.02
Bonaire				83.48	77.40	38.85	199.73	1515.60
Bosnia and Her	0.780	0.718	5.000	28.80	25.80	3.70	58.30	5062.27
Botswana	0.693	-	-	-	-	-	-	1064.52
Brazil	0.754	0.686	6.780	88.70	82.30	57.70	228.70	3260.90
British Virgin				64.88	60.39	12.32	137.59	2042.64
Brunei	0.829	0.704		100.00	100.00	77.83	277.83	363.03
Bulgaria	0.795	0.805	6.530	30.40	30.00	11.10	71.50	5675.94
Burkina Faso	0.449	0.286	3.080	20.90	16.00	0.00	36.90	17.55
Burundi	0.426	0.424	2.130	0.20	0.20	0.00	0.40	1.16
Cape Verde	0.662	-	7.650	64.20	55.50	0.10	119.80	701.33
Cambodia	0.593	0.487	3.180	91.10	87.30	62.10	240.50	182.25
Cameroon	0.576	0.547	2.560	12.20	10.30	1.50	24.00	70.72
Canada	0.935	0.899	8.880	91.00	83.30	78.60	252.90	1379.45
Cayman Islands	-	-	-	94.47	92.31	36.62	223.40	538.4
Central Africa	0.576	0.341	1.350	40.60	40.10	4.40	85.10	20.25
Chad	0.394	0.298	1.670	22.80	22.00	0.00	44.80	10.95
Chile	0.855	0.800	8.220	94.60	92.60	82.48	269.68	3159.45
China	0.768	0.644	1.940	93.20	90.90	58.20	242.30	85.47
Colombia	0.752	0.676	6.720	84.30	72.40	28.40	185.10	2751.41
Comoros	0.558	0.473	3.200	50.50	45.70	0.00	96.20	191.25
Democratic Republic of Congo	0.479	0.496	1.480	7.30	6.20	0.00	13.50	14.83
Republic of the Congo	0.571	0.526	2.790	12.60	11.90	0.00	24.50	65.15
Cook Islands				86.04	83.85	58.30	228.19	117.43
Costa Rica	0.809	0.719	8.290	89.90	84.10	54.20	228.20	1808.20
Ivory Coast	0.550	0.424	4.220	46.80	39.50	7.70	94.00	29.65
Croatia	0.858	0.791	6.500	57.30	55.50	24.60	137.40	4588.92
Cuba	0.764	0.780	2.650	94.60	88.30	76.50	259.40	760.78
Curaçao							0.00	
Cyprus	0.896	0.808	7.380	75.60	72.80	54.10	202.50	1522.31
Czech Republic	0.889	0.893	7.970	65.20	64.40	48.00	177.60	4099.39
Denmark	0.948	0.920	9.280	82.30	81.70	62.80	226.80	1500.1
Djibouti	0.509	0.309	2.740	32.10	30.10	0.00	62.20	168.62
Dominica	0.720	0.613		45.83	42.89	5.55	94.27	1017.07
Dominican Republic	0.767	0.643	6.390	67.30	56.00	23.20	146.50	390.42
Ecuador	0.740	0.697	5.690	86.80	80.60	58.60	226.00	2001.78
Egypt	0.731	0.604	2.930	52.50	39.10	9.60	101.20	223.46
El Salvador	0.675	0.580	5.050	71.70	67.50	35.30	174.50	667.57
Equatorial Guinea	0.596	0.443	1.920	19.30	15.30	0.30	34.90	109.26
Eritrea	0.492	0.281	2.030	-	-	-	-	27.96
Estonia	0.890	0.869	7.960	65.20	63.70	44.20	173.10	2191.45
Eswatini	0.597	-	3.010	42.40	42.30	12.70	97.40	1187.50
Ethiopia	0.498	0.327	3.170	37.50	31.90	2.10	71.50	61.39
Fiji	0.730	0.785	5.550	79.40	71.50	19.00	169.90	951.85
Faroe Islands	-	-	-	85.37	83.69	43.56	212.62	527.14
Finland	0.940	0.905	9.290	81.80	78.60	77.20	237.60	1960.75
France	0.903	0.840	8.070	81.10	78.80	69.00	228.90	2599.32
French Guiana	-	-	-	33.58	30.29	15.51	79.38	1356.02
French Polynesia	-	-	-	67.96	66.55	36.42	170.93	2118.89
Gabon	0.706	0.628	3.400	14.00	11.60	0.10	25.70	128.51
The Gambia	0.500	0.372	4.470	38.70	18.80	1.30	58.80	137.47
Georgia	0.802	0.845	5.200	44.50	34.40	6.70	85.60	4575.38
Germany	0.942	0.940	8.800	77.90	76.30	77.20	231.40	2098.83
Gibraltar	-	-	-	100.00	100.00	124.00	324.00	336.28
Ghana	0.632	0.558	6.430	38.70	28.90	8.20	75.80	43.67
Greece	0.887	0.838	7.970	74.00	71.30	65.40	210.70	3646.52
Greenland	-	-	-	76.62	67.83	0.00	144.45	371.72
Grenada	0.795	0.758	-	39.20	34.69	6.59	80.48	1897.03
Guatemala	0.627	0.514	4.680	52.50	41.70	24.20	118.40	1132.10
Guinea	0.465	0.339	2.320	46.40	25.10	1.70	73.20	33.7
Guinea-Bissau	0.483	0.392	2.560	39.40	21.70	0.05	61.15	84.06
Guyana	0.714	0.596	6.340	62.70	48.30	10.20	121.20	1590.15
Haiti	0.535	0.433	2.810	3.50	2.10	0.00	5.60	74.23
Honduras	0.621	0.502	5.150	65.10	58.10	46.30	169.50	1065.29
Hungary	0.846	0.815	6.640	65.90	63.70	44.20	173.80	4900.82
Iceland	0.959	0.912	9.520	84.50	81.30	69.30	235.10	498.79
India	0.633	0.556	7.040	74.40	68.90	16.10	159.40	376.31
Indonesia	0.705	0.622	6.710	74.40	63.00	24.20	161.60	587.72
Iran	0.774	0.741	1.960	77.50	69.70	37.10	184.30	1656.74
Iraq	0.686	0.534	3.130	28.20	19.70	0.70	48.60	570.27
Republic of Ireland	0.945	0.918	9.130	82.20	81.20	58.60	222.00	1864.58
Israel	0.919	0.874	7.930	72.90	66.80	54.90	194.60	1344.08
Italy	0.895	0.791	7.690	85.40	80.60	77.20	243.20	3167.57
Jamaica	0.709	0.690	7.130	28.50	25.10	1.50	55.10	1269.02
Japan	0.925	0.848	8.330	83.10	81.90	68.44	233.44	602.61
Jordan	0.720	0.711	3.170	47.30	44.70	6.70	98.70	1251.3
Kazakhstan	0.811	0.814	3.080	57.80	56.60	35.40	149.80	983.19
Kenya	0.575	0.551	5.050	26.30	20.00	3.10	49.40	105.3
Kiribati	0.624	0.620	-	-	-	-	-	99.06
South Korea	0.925	0.862	8.030	87.20	86.30	79.80	253.30	693.5
Kuwait	0.831	0.620	3.830	80.90	78.30	34.10	193.30	602.03
Kyrgyzstan	0.692	0.735	3.620	24.80	20.60	3.50	48.90	154.44
Laos	0.607	0.484	1.770	83.70	74.50	27.90	186.10	89.12
Latvia	0.863	0.866	7.370	70.80	68.70	29.00	168.50	4005.07
Lebanon	0.706	0.637	3.640	40.10	35.40	9.70	85.20	1994.08
Lesotho	0.514	0.502	6.190	46.50	43.20	6.30	96.00	307.48
Liberia	0.481	0.434	5.430	75.60	72.60	0.04	148.24	55.44
Libya	0.718	0.616	2.060	33.60	17.90	2.60	54.10	944.9
Liechtenstein	0.935	0.827	-	68.86	68.00	47.25	184.11	2210.65
Lithuania	0.875	0.879	7.310	70.00	67.30	33.80	171.10	3547.38
Luxembourg	0.930	0.792	8.810	76.20	73.20	72.00	221.40	1544.16
Macau	-	-	-	-	-	-	-	-
Madagascar	0.501	0.498	5.700	7.20	6.90	0.40	14.50	48.16
Malawi	0.512	0.451	5.910	20.50	16.20	2.80	39.50	131.63
Malaysia	0.803	0.719	7.300	86.90	85.10	50.30	222.30	1095.84
Maldives	0.747	0.560		73.80	71.20	30.90	175.90	603.29
Mali	0.428	0.298	3.230	15.30	12.30	0.00	27.60	32.88
Malta	0.918	0.818	7.700	92.70	91.30	89.30	273.30	1659.50
Marshall Island	0.639	0.723	-	-	-	-	-	408.72
Martinique	-	-	-	40.18	38.78	24.76	103.72	3003.98
Mauritania	0.556	0.389	4.030	44.20	32.10	7.90	84.20	210.51
Mauritius	0.802	0.729	8.140	88.80	86.00	50.50	225.30	811.86
Mayotte	-	-	-	-	-	-	-	573.42
Mexico	0.758	0.678	5.250	76.80	64.10	44.20	185.10	2626.64
Federated States of Micronesia	0.628	0.590	-	72.90	70.34	30.50	173.74	569.29
Moldova	0.767	0.710	6.230	40.40	39.50	14.00	93.90	3713.42
Monaco	-	-	-	69.02	67.96	48.54	185.52	1835.07
Mongolia	0.739	0.766	6.350	69.70	66.60	32.20	168.50	672.09
Montenegro	0.832	0.790	6.450	47.10	45.80	16.30	109.20	4232.30
Montserrat	-	-	-	42.09	39.05	11.26	92.40	1812.83
Morocco	0.683	0.529	5.040	67.80	63.70	18.70	150.20	435.07
Mozambique	0.446	0.385	3.510	59.80	56.80	3.00	119.60	68.25
Myanmar	0.585	0.443	0.740	63.80	50.60	4.10	118.50	359.81
Nauru	-	-	-	100.00	100.00	71.12	271.12	78.8
Namibia	0.615	0.571	6.520	23.60	20.50	4.00	48.10	1597.96
Nepal	0.602	0.502	4.490	94.30	82.20	30.00	206.50	393.85
Netherlands	0.941	0.906	9.000	73.30	68.60	69.20	211.10	1308.7
New Zealand	0.937	0.917	9.610	84.60	81.40	68.40	234.40	679.23
Nicaragua	0.667	0.558	2.500	93.00	89.30	42.60	224.90	35.26
Niger	0.400	0.214	3.730	24.50	21.40	0.00	45.90	12.2
Nigeria	0.535	0.483	3.730	31.00	24.90	3.00	58.90	14.4
North Macedonia	0.770	0.691	6.100	41.00	40.20	7.80	89.00	4756.39
Norway	0.961	0.915	9.810	80.80	75.50	69.60	225.90	1054.78
Oman	0.816	0.706	3.120	63.80	59.70	15.30	138.80	1011.3
Pakistan	0.544	0.411	4.130	63.20	59.90	22.20	145.30	130.00
Palau	0.767	0.844	-	100.00	100.00	71.20	271.20	497.68
Palestine	0.715	0.660	3.860	41.90	37.00	7.00	85.90	1087.22
Panama	0.805	0.692	6.910	81.50	73.30	46.50	201.30	1962.31
Papua New Guinea	0.558	0.430	5.970	4.10	3.40	0.30	7.80	66.06
Paraguay	0.717	0.631	5.890	55.70	49.50	27.80	133.00	2939.20
Peru	0.762	0.689	5.920	91.50	86.10	84.70	262.30	6507.1
Philippines	0.699	0.661	6.730	71.40	67.30	19.20	157.90	577.59
Poland	0.876	0.866	7.040	60.20	59.60	39.90	159.70	3004.55
Portugal	0.866	0.759	7.950	94.80	86.20	68.10	249.10	2697.15
Puerto Rico	-	-	-	100.00	93.97	62.23	256.20	1825.72
Qatar	0.855	0.698	3.650	98.90	98.90	65.90	263.70	256.02
Réunion	-	-	-	-	-	-	-	945.52
Romania	0.821	0.762	6.450	42.40	42.20	9.10	93.70	3488.94
Russia	0.822	0.832	2.280	60.80	54.50	12.80	128.10	2767.46
Rwanda	0.534	0.450	3.100	81.60	71.60	45.40	198.60	106.58
Saint Kitts and Nevis	0.777	0.680	-	63.53	50.76	6.51	120.80	964.75
Collectivity of Saint Martin	-	-	-	-	-	-	-	1445.31
Saint Lucia	0.715	0.676	-	32.75	38.78	5.55	77.08	2279.40
Saint Vincent	0.751	0.655	-	-	-	-	-	1192.78
Samoa	0.707	0.692	-	-	-	-	-	130.4
San Marino	0.853		-	76.20	68.59	56.16	200.95	3739.98
São Tomé and Principe	0.618	0.557	-	58.10	47.30	14.90	120.30	351.91
Saudi Arabia	0.875	0.787	2.080	77.40	72.80	45.30	195.50	264.94
Senegal	0.511	0.367	5.720	11.60	8.10	3.70	23.40	113.82
Serbia	0.802	0.778	6.330	49.20	48.10	27.50	124.80	2627.79
Seychelles	0.785	0.727		90.10	84.80	45.69	220.59	1605.45
Sierra Leone	0.477	0.390	5.030	48.10	37.90	1.10	87.10	14.53
Singapore	0.939	0.832	6.220	90.70	90.00	78.10	258.80	342.91
Slovakia	0.848	0.832	7.070	52.00	51.10	30.90	134.00	3750.72
Slovenia	0.918	0.886	7.750	60.30	58.20	31.30	149.80	4,475.33
Solomon Island	0.564	0.469		50.10	37.00	4.00	91.10	274.76
Somalia	-	-	-	56.45	48.24	0.18	104.87	77.34
South Africa	0.713	0.708	7.050	37.90	33.00	6.50	77.40	1712.95
South Sudan	0.385	0.297		19.30	18.90	0.00	38.20	12.64
Spain	0.905	0.824	8.070	87.20	85.90	54.90	228.00	2562.14
Sri Lanka	0.782	0.749	6.470	78.20	67.30	37.50	183.00	773.45
Sudan	0.508	0.328	2.470	24.00	18.90	2.10	45.00	107.65
Suriname	0.730	0.636	6.950	45.70	40.50	8.30	94.50	2271.68
Sweden	0.947	0.904	9.390	75.50	73.70	93.30	242.50	2401.29
Switzerland	0.962	0.897	9.140	70.60	69.60	55.80	196.00	1611.58
Syria	0.577	0.412	1.430	17.40	12.00	0.40	29.80	142.96
Taiwan	-	-	-	92.50	87.40	93.30	273.20	635
Tajikistan	0.685	0.659	1.940	55.40	53.70	51.90	161.00	12.56
Tanzania	0.549	0.441	5.100	48.30	46.00	12.00	106.30	12.92
Thailand	0.800	0.661	6.670	82.20	77.30	46.10	205.60	481.08
East Timor	0.607	0.505	7.060	66.30	59.20	22.50	148.00	102.89
Togo	0.539	0.506	2.990	27.10	17.60	5.20	49.90	32.77
Tonga	0.745	0.770	-	-	-	-	-	112.29
Trinidad and Tobago	0.810	0.722	7.160	53.90	51.30	12.10	117.30	2867.33
Tunisia	0.731	0.659	5.510	61.10	54.10	10.60	125.80	2381.25
Turkey	0.838	0.639	4.350	68.70	63.10	49.10	180.90	1188.39
Turkmenistan	0.745	0.626	1.660	61.80	61.20	55.40	178.40	-
Tuvalu	0.641	-	-	82.90	80.61	48.54	212.05	88.22
Uganda	0.525	0.525	4.550	41.20	28.30	0.90	70.40	76.83
Ukraine	0.773	0.794	5.420	35.70	34.30	1.70	71.70	2768.59
United Arab Emirates	0.911	0.738	2.900	77.40	72.80	45.30	195.50	248.81
United Kingdom	0.929	0.914	8.280	92.90	86.90	69.00	248.80	3438.24
United States	0.921	0.903	7.850	80.76	68.87	35.23	184.86	3384.31
Uruguay	0.809	0.733	8.910	86.50	83.30	82.30	252.10	2236.77
Uzbekistan	0.727	0.718	2.120	62.10	51.20	42.20	155.50	29.34
Vanuatu	0.607	0.529	-	47.20	42.90	5.50	95.60	42.85
Venezuela	0.691	0.741	2.230	77.90	52.60	2.30	132.80	206.91
Vietnam	0.703	0.626	2.730	92.80	87.60	58.90	239.30	440.04
Western Sahara	-	-	-	-	-	-	-	-
Yemen	0.455	0.349	1.950	3.40	2.50	0.20	6.10	64.07
Zambia	0.565	0.580	5.800	59.50	46.50	5.50	111.50	203.27
Zimbabwe	0.593	0.558	2.920	44.10	33.20	8.10	85.40	350.91

### Countries and territories

The countries and territories were split into the following geographic groups: North America (NA), South America (SA), Western Europe (WE), Eastern Europe (EE), Asia, Middle East (ME), Africa, and Australia.

### Statistical analysis

The statistical analysis was done with SPSS 26 to determine the strength of correlation via Pearson’s correlation and linear regression analysis for scale variables, eta coefficient test for categorical and scale variables, mean, confidence interval (CI)– 95%, p values (< 0.05), etc.

## Results

### General Information

**Fig. 1:**
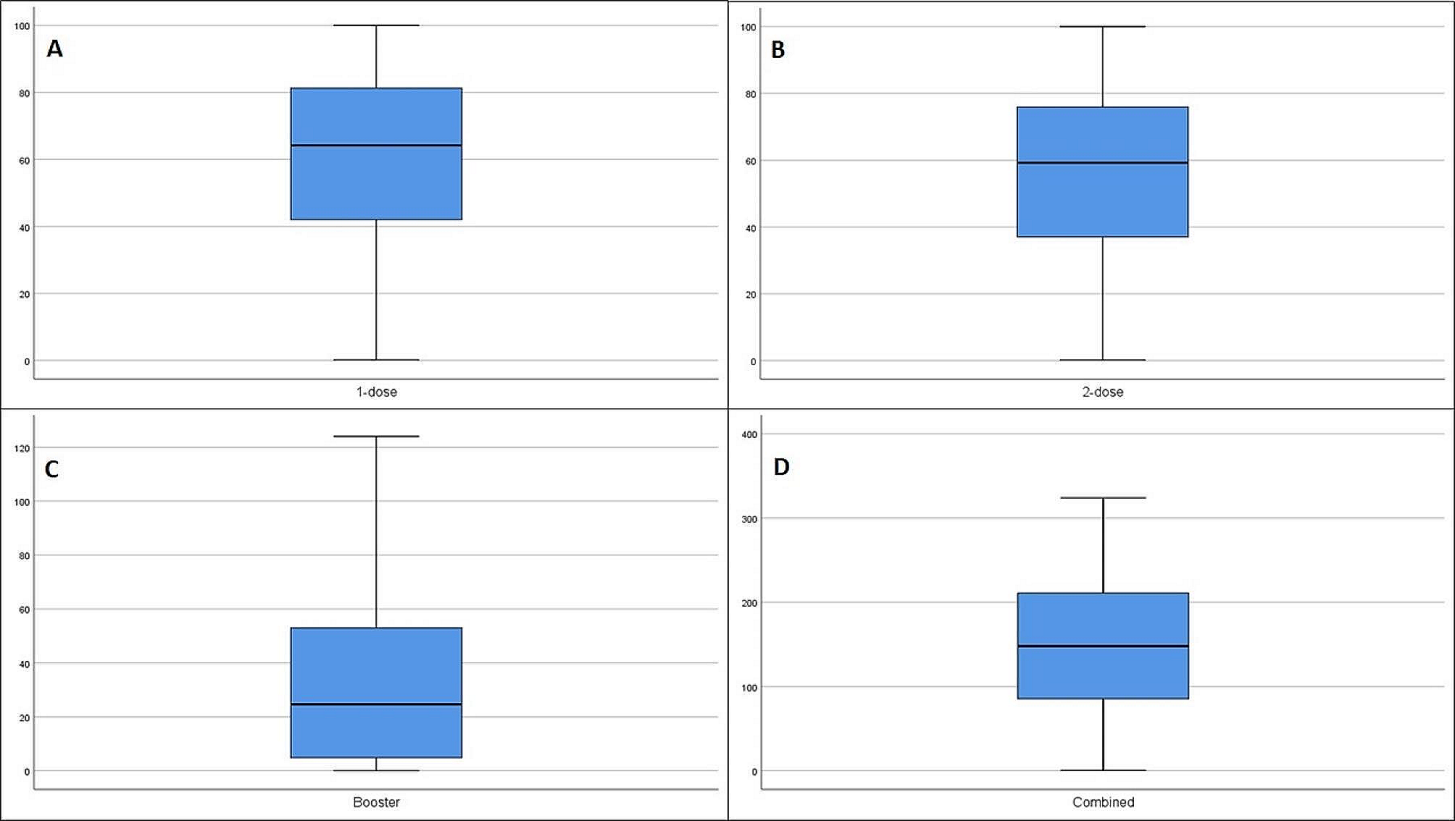
This figure depicts worldwide results of 1-dose (**A**), 2-dose (**B**), booster (**C**) and combined (**D**) vaccination rates (in percentages)

The worldwide mean for 1-dose, 2-dose, booster dose and combined vaccination rate were, respectively, 60.59% (57.11–64.08, CI– 95%), 55.58% (52.05–59.10, CI– 95%), 30.66% (26.76–34.57, CI– 95%), 146.84% (136.39-157.29, CI– 95%) (Fig. 1).

**Fig. 2:**
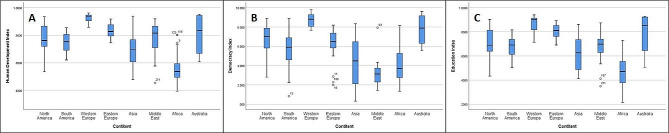
This figure depicts results of HDI (**A**), EI (**B**), and DI (**C**) vaccination rates in relation to the geographic location

We also calculated mean HDI, EI and DI for separate geographic locations (Fig. 2) and they were as follows:

**HDI** for NA– 0.770 (0.664–0.876, CI– 95%), SA– 0.744 (0.712–0.776, CI– 95%), WE– 0.92 (0.911–0.942, CI– 95%), EE– 0.832 (0.811–0.852, CI– 95%), Asia– 0.702 (0.653–0.752, CI– 95%), ME– 0.770 (0.698–0.841, CI– 95%), Africa– 0.559 (0.530–0.589, CI– 95%), Australia– 0.806 (0.540–1.071, CI– 95%).

**EI** for NA − 0.701 (0.574–0.827, CI– 95%), SA– 0.679 (0.637–0.722, CI– 95%), WE– 0.864 (0.831–0.897, CI– 95%), EE– 0.806 (0.782–0.830, CI– 95%), Asia– 0.620 (0.559–0.680, CI– 95%), ME– 0.657 (0.581–0.734, CI– 95%), Africa– 0.470 (0.434–0.506, CI– 95%), Australia– 0.784 (0.470–1.097, CI– 95%);

**DI** for NA– 6.64 (5.06–8.22, CI– 95%), SA– 5.56 (4.55–6.57, CI– 95%), WE– 8.64 (8.29-9.00, CI– 95%), EE– 6.24 (5.56–6.91, CI– 95%), Asia– 4.47 (3.48–5.45, CI– 95%), ME– 3.30 (2.45–4.14, CI– 95%), Africa– 4.00 (3.51–4.48, CI– 95%), Australia– 7.73 (4.87–10.59, CI– 95%).

Either weak or very weak correlation was observed between Covid-19 death rate per 1-million population and 1-dose, 2-dose, booster, and combined vaccination rate– r (196) = 0.165, *p* < 0.001, r (196) = 0.200, *p* < 0.001, r (196) = 234, *p* < 0.001, and r(196) = 0.210, *p* < 0.001, respectively. This leads us to believe that higher death rates, unfortunately, in most of the countries, did not act as a stimuli to get a vaccine shot.

### Education Index and COVID-19 vaccination rate

**Fig. 3:**
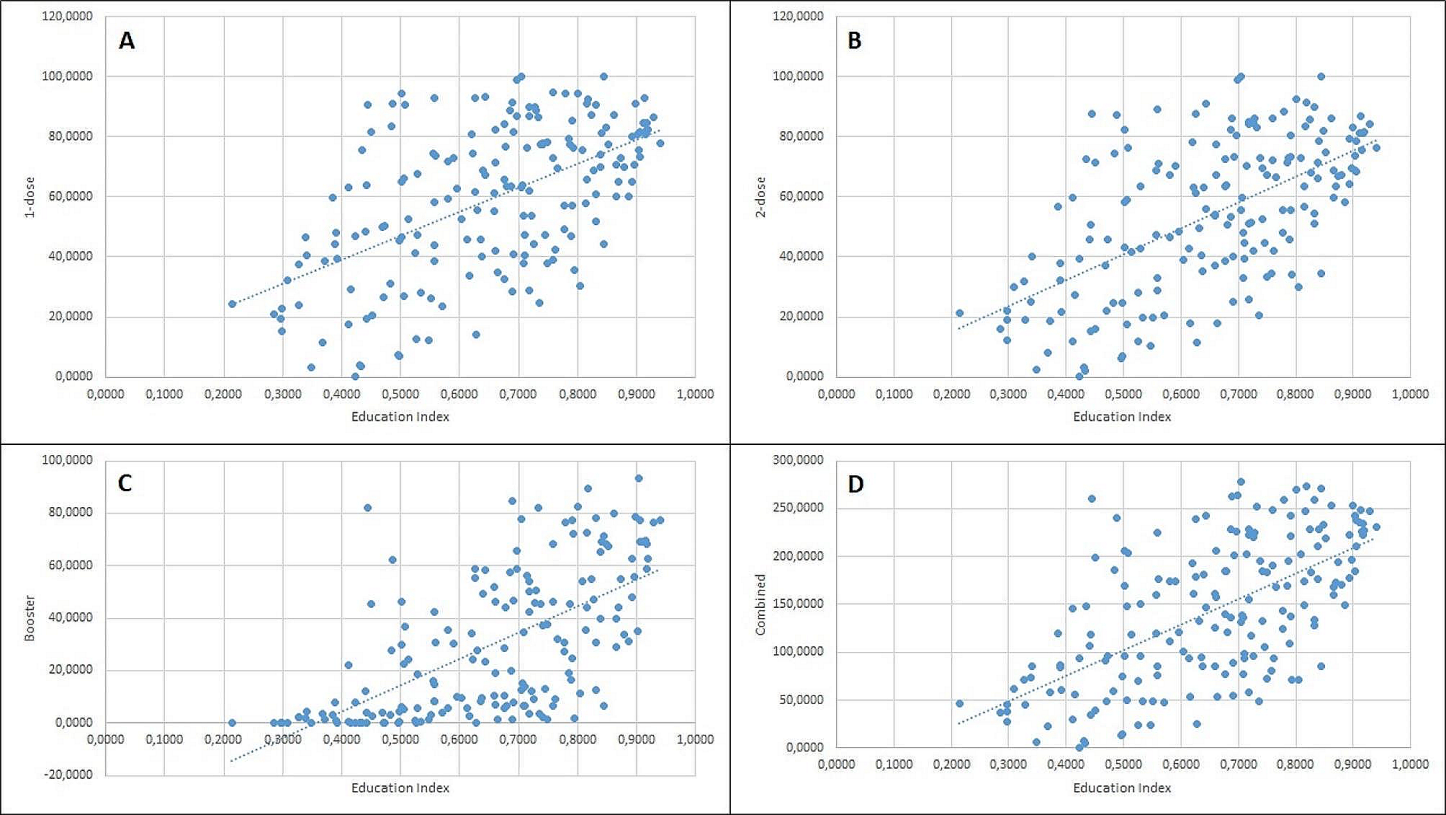
Figure 1 This figure depicts linear relationship between 1-dose (**A**), 2-dose (**B**), booster (**C**) and combined (**D**) vaccination rates (in percentages) and EI.

A Pearson correlation coefficient and linear regression analysis were computed to assess the linear relationship between EI and vaccination percentages of 1-dose, 2-dose, booster, as well as combined values (Fig. 3). There was a positive correlation between EI and 1-dose vaccination rate, r (177) = 0.560, r-squared– 0.313, F– 80.66, Beta– 80.19 (62.57–97.82, CI– 95%), *p* < 0.001, There was a positive correlation between EI and 2-dose vaccination rate, r (177) = 0.599, r-squared– 0.359, F– 99.22, Beta– 86.59 (69.43-103.74, CI– 95%), *p* < 0.001. There was a positive correlation between EI and booster dose rate, r (177) = 0.642, r-squared– 0.412, F– 123.91, Beta– 100.50 (82.68-118.31, CI– 95%), *p* < 0.001. There was a positive correlation between EI and combined vaccination rate, r (177) = 0.626, r-squared– 0.392, F– 114.02, Beta– 267.29 (217.89-316.68, CI– 95%), *p* < 0.001.

### Human Development insights and COVID-19 vaccination rate

**Fig. 4:**
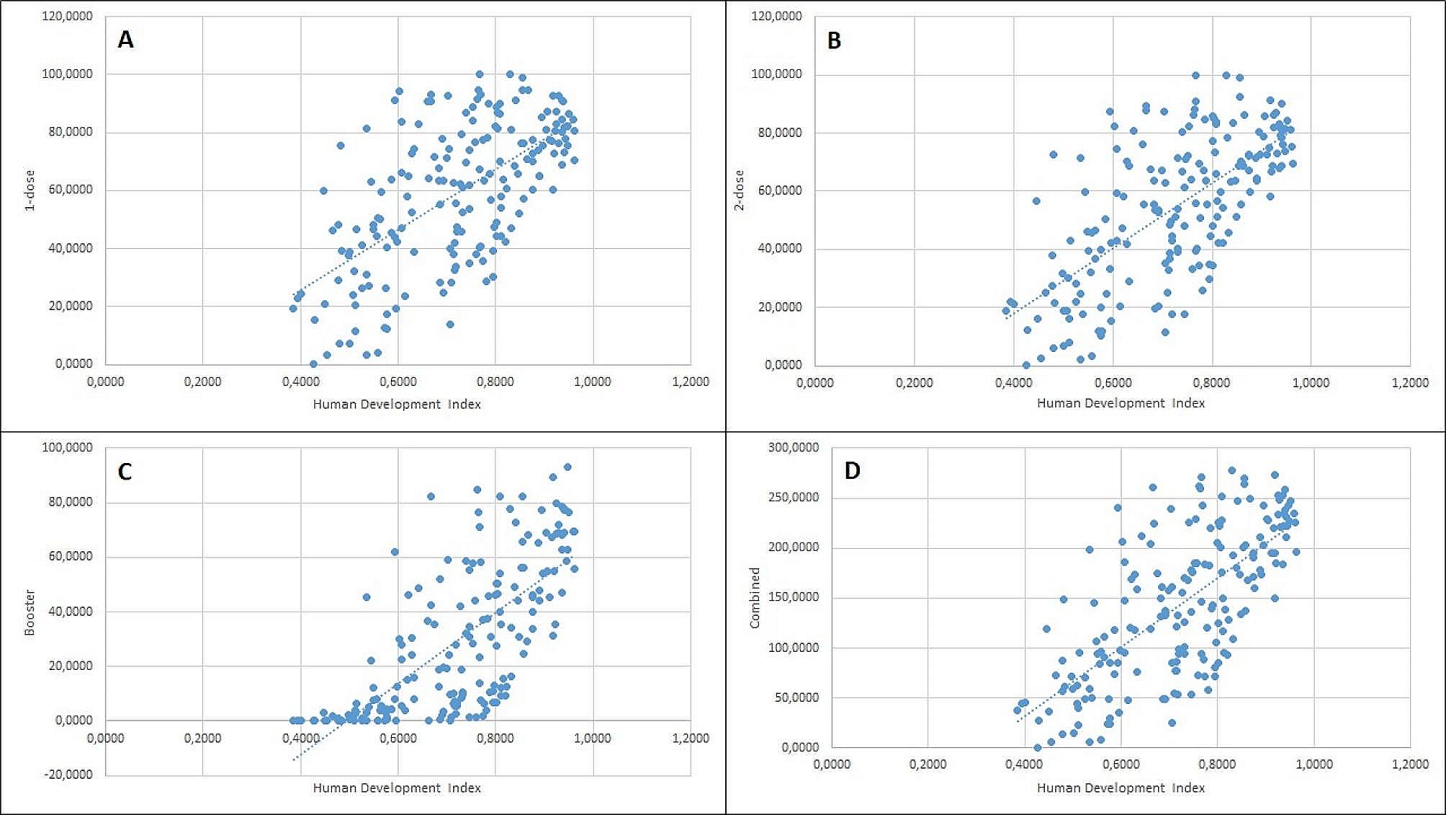
This figure depicts linear relationship between 1-dose (**A**), 2-dose (**B**), booster (**C**) and combined (**D**) vaccination rates (in percentages) and HDI.

A Pearson correlation coefficient and linear regression analysis were computed to assess the linear relationship between HDI and vaccination percentages of 1-dose, 2-dose, booster, as well as combined values (Fig. 4). There was a positive correlation between HDI and 1-dose vaccination rate, r (181) = 0.632, r-squared– 0.399, F– 120.10, Beta– 104.37 (85.57-123.16, CI– 95%), *p* < 0.001. There was a positive correlation between HDI and 2-dose vaccination rate, r (181) = 0.671, r-squared– 0.451, F– 120.10, Beta– 111.76 (93.66-129.86, CI– 95%), *p* < 0.001. There was a positive correlation between HDI and booster dose rate, r (181) = 0.718, r-squared– 0.516, F– 192.87, Beta– 130.10 (111.61-148.58, CI– 95%), *p* < 0.001. There was a positive correlation between HDI and combined vaccination rate, r (181) = 0.703, r-squared– 0.494, F– 176.92, Beta– 346.23 (294.87-397.59, CI– 95%), *p* < 0.001.

### Democracy Index and COVID-19 vaccination rate

**Fig. 5:**
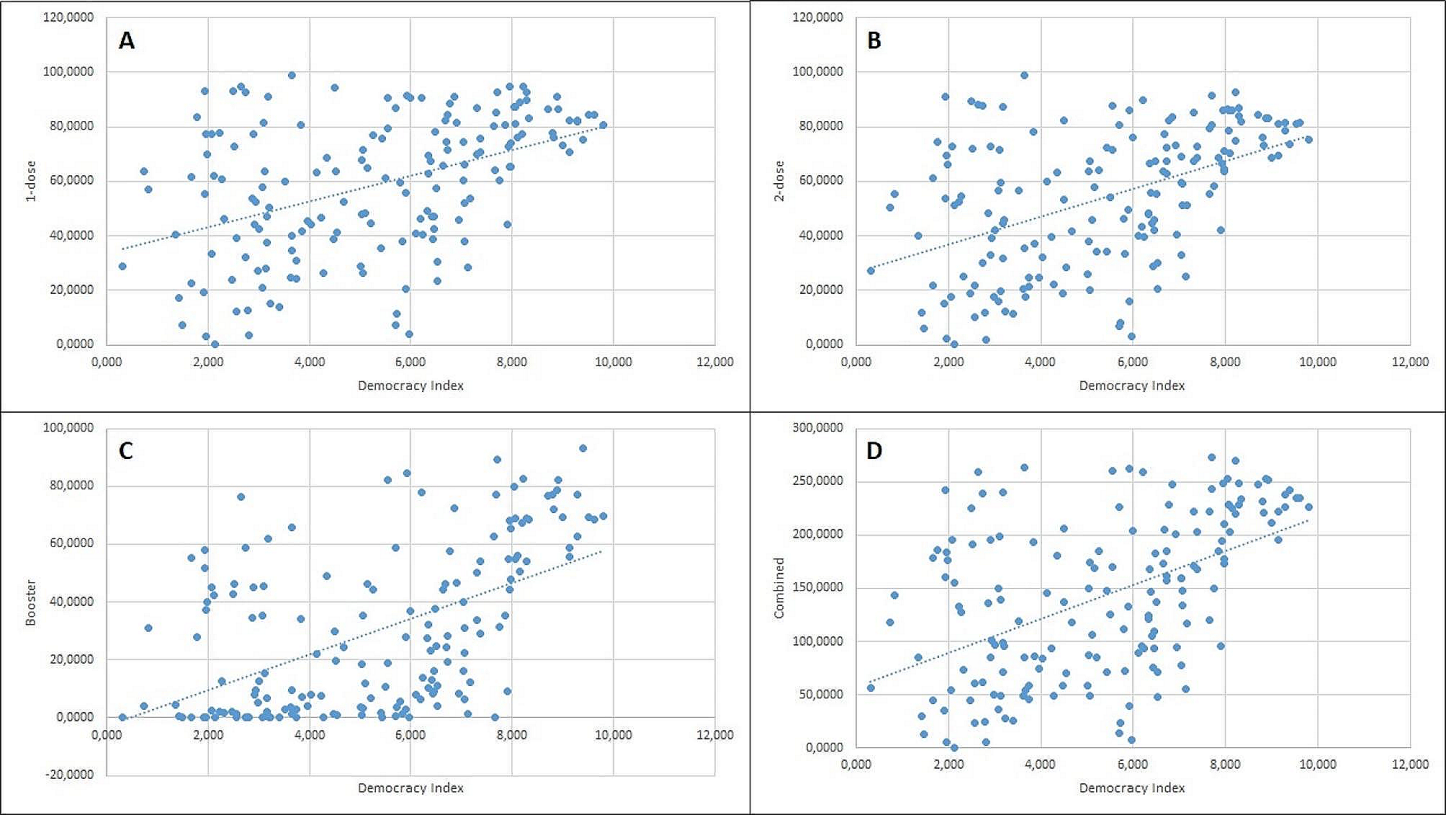
This figure depicts linear relationship between 1-dose (**A**), 2-dose (**B**), booster (**C**) and combined (**D**) vaccination rates (in percentages) and DI.

A Pearson correlation coefficient and linear regression analysis were computed to assess the linear relationship between DI and vaccination percentages of 1-dose, 2-dose, booster, as well as combined values (Fig. 5). There was a positive correlation between DI and 1-dose vaccination rate, r (163) = 0.445, r-squared– 0.198, F– 40.23, Beta– 4.70 (3.23–6.16, CI– 95%), *p* < 0.001. There was a positive correlation between DI and 2-dose vaccination rate, r (163) = 0.479, r-squared– 0.230, F– 48.62, Beta– 5.09 (3.651–6.53, CI– 95%), *p* < 0.001. There was a positive correlation between DI and booster dose rate, r (163) = 0.534, r-squared– 0.285, F– 65.12, Beta– 6.18 (4.67–7.69, CI– 95%), *p* < 0.001. There was a positive correlation between DI and combined vaccination rate, r (163) = 0.508, r-squared– 0.258, F– 56.64, Beta– 15.98 (11.78-20.173, CI– 95%), *p* < 0.001.

### Geographic location and Covid-19 vaccination rate

**Fig. 6:**
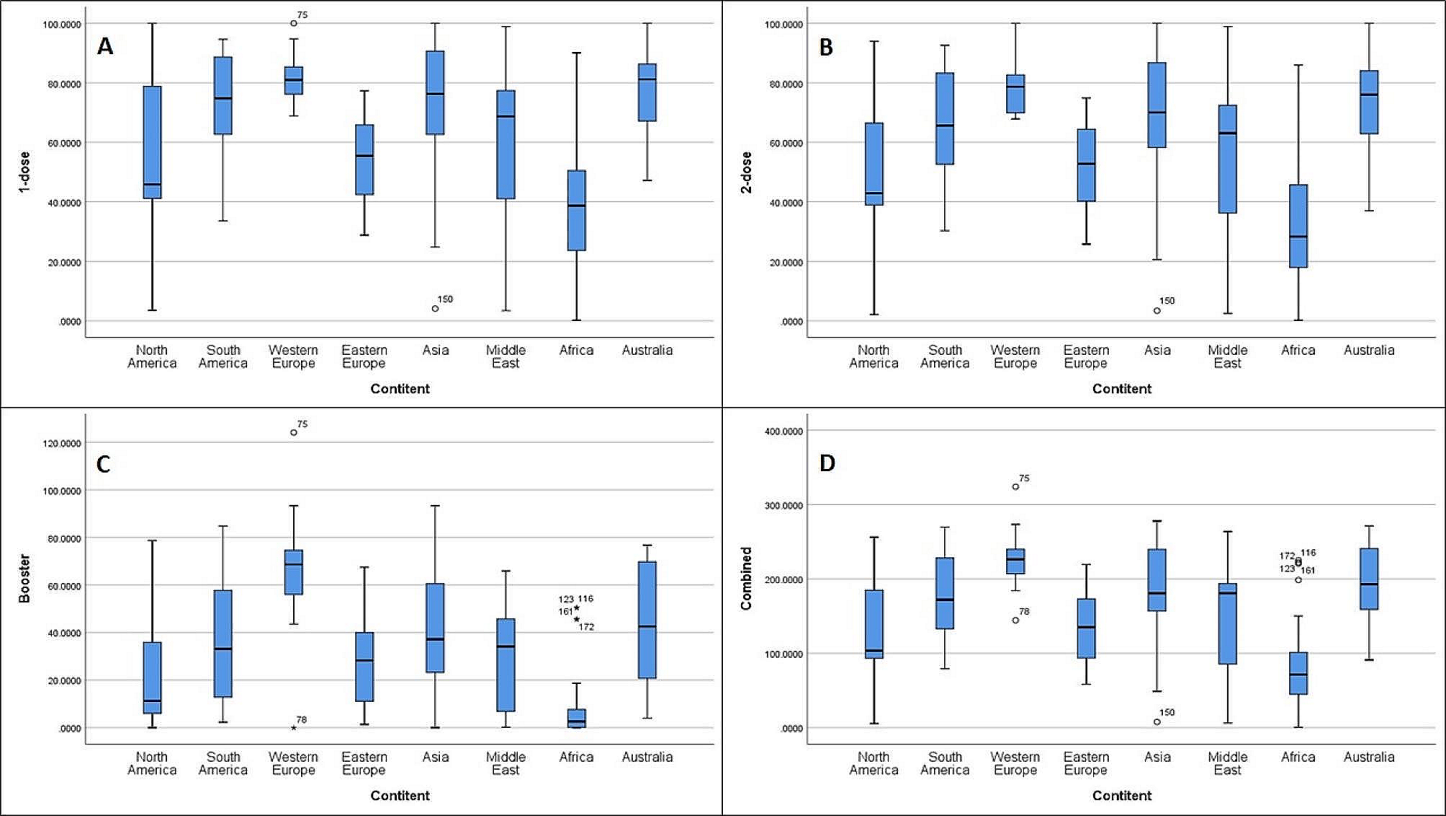
This figure depicts results of 1-dose (**A**), 2-dose (**B**), booster (**C**) and combined (**D**) vaccination rates (in percentages) in relation to the geographic location

**Fig. 7:**
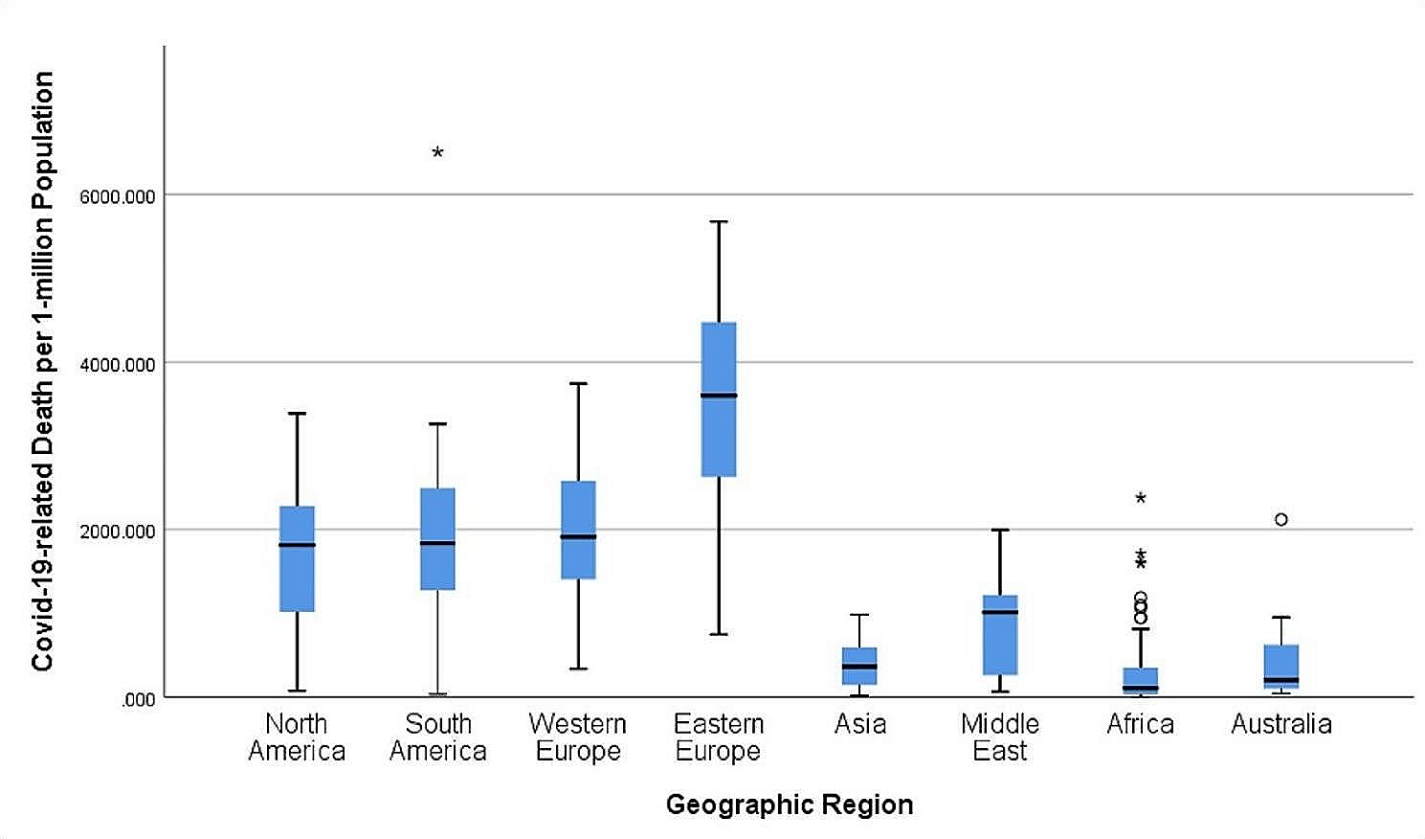
This figure depicts COVID-19 related death per 1-milion population distribution in various geographic locations

We tried to determine if there were different results regarding vaccination rate, COVID-19 related death results in various geographic location (Figs. 6 and 7).

The 1-dose vaccination rate mean for NA was 57.11% (41.80-72.42, CI– 95%), SA– 72.79% (65.68–79.91, CI– 95%), WE– 81.31% (77.9-84.73, CI– 95%), EE– 54.73% (48.90-60.55, CI– 95%), Asia– 72.71 (63.84–81.58, CI– 95%), ME– 57.91% (43.01–72.8, CI– 95%), Africa– 39.73 (33.73–45.72, CI– 95%), Australia– 77.00% (66.23–87.76, CI– 95%).

The 2-dose vaccination rate mean for NA was 51.9% (37.97–65.83, CI– 95%), SA– 65.99% (58.72–73.25, CI– 95%), WE– 78.12% (74.59–81.5, CI– 95%), EE– 52.24% (46.4-58.09, CI– 95%), Asia– 67.79% (59.08–76.50, CI– 95%), ME– 53.7% (38.54–68.85, CI– 95%), Africa– 33.04% (27.21–38.86, CI– 95%), Australia– 73.13 (60.61–85.66, CI– 95%).

The booster dose vaccination rate mean for NA was 23.52% (10.42–36.61), SA– 38.15% (27.59–48.71, CI– 95%), WE– 65.45 (56.17–74.74, CI– 95%), EE– 27.40 (19.65–35.16, CI– 95%), Asia– 41.95 (31.62–52.29, CI– 95%), ME– 27.85% (15.18–40.51, CI– 95%), Africa– 6.97% (3.50-10.44, CI– 95%), Australia– 42.68% (25.81–59.55, CI– 95%).

The combined vaccination rate mean for NA was 132.54% (91.80-173.28, CI– 95%), SA– 176.94% (153.38–200.50, CI– 95%), WE– 224.90% (210.63-239.17, CI– 95%), EE– 134.38% (115.51-153.26, CI– 95%), Asia– 182.46% (156.35-208.57, CI– 95%), ME– 139.46% (97.71-181.22, CI– 95%), Africa– 79.75% (65.26–94.23, CI– 95%), Australia– 192.81% (153.65-231.98, CI– 95%).

Eta coefficient was computed to assess the relationship between geographic location and different vaccination rates (vaccination rate was a dependent variable, while the geographic location was independent). 1-dose vaccination rate– η = 0.610 *p* < 0.001, 2-dose vaccination rate– η = 0.633 *p* < 0.001, booster dose– η = 0.657, *p* < 0.001, combined - η = 0.645, *p* < 0.001.

**Fig. 8:**
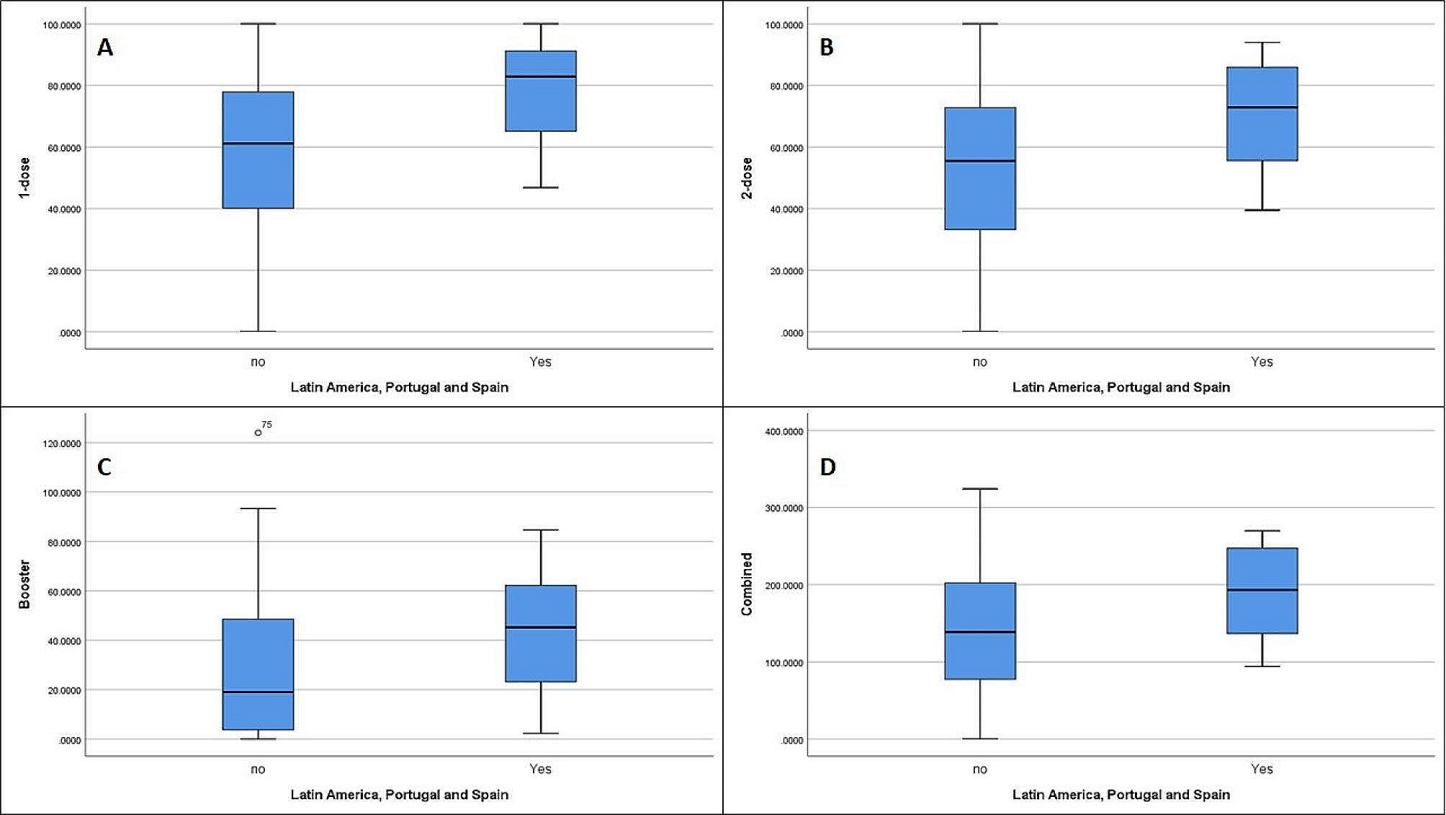
This figure compares the results from Latin America, Spain, and Portugal to the results from the rest of the world in regards to 1-dose (**A**), 2-dose (**B**), booster (**C**) and combined (**D**) vaccination rates (in percentages)

It caught our attention that Spanish and Portuguese speaking countries had good vaccination results, hence, we grouped Spain, Portugal, and Latin American countries together and ran the analysis. They were close to the WE mean vaccination values and much higher than EE results, whilst the HDI, EI and DI of EE were much closer to WE (Fig. 2). So, we took these countries and compared them to the rest of the world (Fig. 8). In these countries the mean 1-dose, 2-dose, booster, and combined vaccination rate was 78.21% (72.12–84.31, CI– 95%), 70.45% (63.58–77.32, CI– 95%), 43.69% (33.37–54.01, CI– 95%), and 192.3% (170.21-214.52, CI– 95%), respectively.

The highest mean for reported COVID-19 related death per 1-million population was observed in EE– 3388.17 (2861.2-3915.15, CI– 95%) (Table 2; Fig. 7).

**Table 2:** The table demonstrates mean, minimum, and maximum values of Covid-19 related death rate in different geographic locations of the world

Geographic Region	Mean	Minimum	Maximum
North America	1700.68294	74.230	3384.310
South America	1963.57370	35.260	6507.100
Western Europe	1913.57458	336.280	3739.980
Eastern Europe	3388.17885	746.520	5675.940
Asia	371.38333	12.560	983.190
Middle East	848.36800	64.070	1994.080
Africa	336.20947	1.160	2381.250
Australia	448.47125	42.850	2118.890
Total	1268.12866	1.160	6507.100

## Discussion

Out of the indicators HDI has the strongest correlation with the vaccination rate, especially the booster doses; Geographic location, EI and DI coming in with the respective order. WE showed the best results. Therefore, according to the findings of the study, if countries continue to progress and manage to increase HDI and EI locally, the vaccine coverage should go up. As the data suggests these indices are in statistically significant relation with higher vaccination rates throughout the world. However, according to the data, there were certain regions that did have lower vaccination rates in relation to their HDI, EI and DI values. The lower vaccination in the EE resulted in the highest reported COVID-19 death per 1-million population when compared to other regions (Table 2; Fig. 7). Therefore, in the discussion we are going to try and isolate all the possible causes for this disparity and elaborate on them, so that we identify possible areas for further investigation.

For example, if we take a closer look at countries in EE, we should sense that there is some other factor that is contributing to the lower vaccination rate, as it showed higher HDI, EI, however, the vaccination rate was lower than in Asia, SA, Latin American Countries, Portugal, and Spain (Figs. 2, 6 and 7). We tried to collect more information on Portuguese/Spanish speaking countries and Asian countries to identify the circumstances that led to better vaccination results than Eastern European countries despite the latter having higher HDI, EI, indices (Figs. 2, 6 and 7).

The low vaccination rate in most countries of Africa can be attributed to logistical issues, which are caused by military conflicts, lack of finances, low accessibility, lack of energy infrastructure, etc. [15, 16].

In certain countries, such as the Russian Federation we do not even know if their official statistics about the vaccination rate can be trusted since there are a lot of reports about fake COVID-19 vaccine certificates [17–19].

The anti-scientific and anti-vaccination campaigns might be the cause for the above, as well as rampant AIDS dissident community, which is built upon the AIDS denialism narratives and they deny the link between HIV and AIDS, promote treatment with natural remedies and lead people to succumb to this terrible disease [20–22].

There is a stark disparity between average indices and vaccination rates in some of the countries in Eastern Europe. Even though there is a general correlation between these indices and COVID-19 vaccination rate, still these countries fell short of hitting the average vaccination milestones.

According to a paper Georgia had delayed immunization campaign, insufficient coordination, little evidence-informed policymaking, no effective measures were taken to tackle disinformation (disinformation mainly was spread by far-right groups having ties with Russian federation), and including politically driven management of the pandemic ended in Georgia having one of the worst vaccination rates and one of the highest death rates per 1-million population [Table 1] [23]. Apart from the low vaccine coverage, the latter can also be attributed to bad patient management, which was mainly caused by bad remote management, due to failure of governmental hot-line, and overload of the healthcare system; The hospitalization rate was up to 15.1%– out of 1 660 249 laboratory-confirmed cases, 250 113 patients had been hospitalized, while a private telehealth clinic managed to maintain significantly lower numbers in regards to hospitalization (0.89% during omicron variant domination and 2.48% during delta variant domination) [24].

Eastern Europe is not the only region where the Russian soft power has been noticed. There have been multiple media reports and scientific studies conducted that has exposed anti-vaccination campaigns conducted and backed by The Russian Federation and China; The main purpose of Russian Federation seems to be undermining western science, spreading misinformation about the pandemic and COVID-19, exacerbating tensions among western society; Although, China and Russia have similarities, the main difference is that China stays consistent with a narrative while Russia is revamping the approach through its firehose of falsehoods strategy [25, 26]. Russian State media– Russia Today, which is followed by many on social platforms such as Twitter, YouTube, and Facebook, was working on amplifying tensions regarding vaccine hesitancy and propagating their country as a global humanitarian and scientific leader, especially galvanizing far right and other fringe groups, such as Proud Boys in the United States or neo-Nazis throughout Europe [26–28], one such case of online bullying and threatening is believed to have ended in suicide of an Austrian doctor [27]. After Center for European Policy Analysis (CEPA) published the study about the Russian and Chinese disinformation, that was a signal to the western countries to increase their efforts to vigorously tackle the disinformation through social media [29]. There have been numerous reports that social media accounts that had previously been spreading vaccine and COVID-19 disinformation shifted their focus and started to spread Russian narrative about the invasion/war in Ukraine, however, another CEPA report shows that this time around the propaganda was not as successful, because various strategies to deter such disinformation were implemented (such as, disinformation and misinformation policy implementation throughout various social media platforms, flagging and taking down misleading information etc.) [28–33]. The information warfare that Russian Federation is waging in the west is not a newly discovered thing and they are engaged not only in undermining medical progress, but electoral campaigns as well [34]; For example, back in 2018 researchers published a paper that studied how Russian Twitter bots and trolls amplified vaccine hesitancy during measles outbreak and, when they did a retrospective review of those accounts, the same trolls and bots were engaged in disseminating divisive topics during 2016 elections, misinformation about 5G wireless technology, in short, they were using everything to create a discord and further polarize the society [34]. All of the above, though, acts as a double-edged sword, since a study from Russia shows that the main reason from vaccine hesitancy are fake news, which creates fear and doubts around the vaccine [33].

Portugal faced a healthcare crisis with overflowing hospitals and soaring COVID-19 deaths, triggering an urgent need to revamp its faltering vaccine program. In response, Vice Adm. Henrique Gouveia e Melo, a former submarine squadron commander, was called upon to lead the charge. Just eight months later, Portugal stands among global vaccination leaders. Portugal’s vaccination success story defied usual challenges. The nation grappled with vaccine misinformation on social media, political divisions, and initial vaccine hesitancy. Admiral Gouveia e Melo, leveraging his military background, instilled a warlike mindset in his team of experts, which comprised military personnel, mathematicians, doctors, and strategists. His key advice for other nations: to select non-political leaders for similar roles. Portugal’s robust national vaccination program, rooted in its experience combating polio, played a pivotal role in its success. The task force, led by Admiral Gouveia e Melo, efficiently designed a system to facilitate mass vaccinations while bolstering public trust by having soldiers and healthcare professionals visibly receive vaccines. Consistency in messaging, emphasizing vaccine safety through diverse personnel, was a crucial element that set Portugal’s vaccination campaign apart from others worldwide [35].

Spain’s decentralization enables customized healthcare solutions, fosters innovation, and enhances responsiveness to regional needs, with the national government playing a facilitating role in coordination rather than imposing centralization. Interestingly, when assessing transparency in COVID-19 health management, Castilla y León, the Basque Country, and the Balearic Islands emerge as the most transparent regions, with rates of 62.5%, 50%, and 50%, respectively. However, it is noteworthy that Asturias, the region with the highest vaccination rates, records a transparency rate of only 40% [36].

According to a study, participants highlighted that belonging to a low-risk group drove vaccine refusal, irrespective of vaccine safety and efficacy. However, higher the risk-group meant higher chance for getting vaccinated [37]. Another crucial factor for vaccine refusal was being religious [37]. Our findings might suggest that since the Pope, Bishops were urging for COVID-19 vaccination in a video message, and were praising scientists for vaccines, calling it an act of love, fostering hope, and stressing the need for widespread availability to end the pandemic, it should have affected the vaccination rates in catholic countries [37, 38].

There are several reasons why COVID-19 vaccination rates were so high in Latin America. One reason is that many Latin American countries have strong public health systems [39, 40]. These public health systems were able to distribute COVID-19 vaccines quickly and efficiently to the population. Another reason is that many Latin Americans have a high level of trust in their governments [41]. This trust was important in ensuring that people were willing to get vaccinated. Finally, many Latin Americans were personally affected by COVID-19. This personal experience made them more likely to get vaccinated [42]. Catholic church being a strong proponent of vaccination must also have been a factor [37, 38].

The most of Asian countries (China, India, Nepal, Thailand etc.) also have higher vaccination rates, relative to their HDI, EI and DI, when compared with EE. Strong public health system, trust of the authorities (highest is China) and cohesive messages from the politicians could have been the reason [43–46].

This study, while insightful, possesses certain constraints that warrant careful consideration for a more comprehensive interpretation of its findings. One noteworthy limitation is the omission of critical factors such as the diverse infrastructural landscapes, logistical capabilities, and varied resource availabilities across the countries and territories. The multifaceted nature of these elements can significantly influence the outcomes and may not be adequately captured within the scope of the study. In addition to these contextual limitations, the study’s reliance on a cross-sectional design introduces inherent challenges. The utilization of non-randomized data, and the inherent difficulty in establishing the directionality of variables in cross-sectional studies poses a substantial challenge to drawing causal inferences. Without a temporal sequence of events, discerning whether certain factors precede or succeed others becomes challenging, limiting the study’s ability to establish causation. Considering these limitations, it is crucial for researchers and policymakers alike to interpret the study’s findings with a nuanced understanding of the contextual intricacies and methodological constraints. Addressing these limitations in future research endeavors will not only enhance the robustness of the conclusions but also contribute to the development of more effective and contextually relevant interventions and policies.

## Conclusion

In conclusion, the data suggests there is a strong positive correlation of COVID-19 vaccination rates with HDI and EI. Vaccine hesitancy is a complex issue, and it goes beyond just considering HDI and EI. The data indicates that factors influencing vaccination rates are diverse and need comprehensive exploration. Thoroughly studying these additional elements is essential to gaining a deeper understanding of the challenges associated with vaccine hesitancy. This multifaceted issue extends beyond these indices, as indicated by diverse factors influencing vaccination rates, particularly evident in regions like Eastern Europe, where there is a lower vaccination rate relative to HDI and EI values as per the results of the study. A multidimensional and evidence-based approach is essential to achieving higher vaccination rates, ultimately saving lives, and mitigating the impact of the future pandemics.

### Electronic supplementary material

Below is the link to the electronic supplementary material.

Supplementary Material 1

## Data Availability

All data generated or analyzed during this study are included in this published article [and its supplementary information files].
